# Risk Factors for Progressive Spinal Sagittal Imbalance in the Short-Term Course after Total Hip Arthroplasty: A 3 Year Follow-Up Study of Female Patients

**DOI:** 10.3390/jcm11175179

**Published:** 2022-09-01

**Authors:** Satoshi Nagatani, Satoru Demura, Satoshi Kato, Tamon Kabata, Yoshitomo Kajino, Noriaki Yokogawa, Daisuke Inoue, Yuki Kurokawa, Motoya Kobayashi, Yohei Yamada, Masafumi Kawai, Hiroyuki Tsuchiya

**Affiliations:** Department of Orthopaedic Surgery, Graduate School of Medical Sciences, Kanazawa University, 13-1 Takara-machi, Kanazawa 920-8641, Japan

**Keywords:** total hip arthroplasty, spinal sagittal imbalance, spinopelvic parameters, sacral slope, sagittal vertical axis, abdominal trunk muscle strength, short-term follow-up, female patients

## Abstract

Total hip arthroplasty (THA) for patients with hip osteoarthritis improves hip flexion contracture, subsequently improving spinal sagittal balance. However, in some cases, spinal sagittal imbalance develops in the course after THA, and its risk factors remain unknown. We aimed to investigate the risk factors of progressive spinal sagittal imbalance after THA. This retrospective cohort study of a prospectively maintained database included female patients aged ≥50 years who underwent THA. Before performing THA, we obtained each patient’s anthropometric and muscle strength measurements and whole-spine radiographs. Three years postoperatively, patients underwent whole-spine radiography to examine changes in the spinal sagittal balance. Patients were assigned into groups on the basis of their preoperative and 3 year postoperative sagittal vertical axis (SVA) values. Patients with 3 year postoperative SVA values ≥40 mm with an increase ≥30 mm were categorized into the imbalance group; the other patients were categorized into the non-imbalance group. Of 103 patients, 11 (10.7%) were in the imbalance group. In multiple logistic regression analysis, preoperative weak abdominal trunk muscle strength (ATMS) (*p* = 0.007) and small sacral slope (SS) (*p* = 0.005) were significant risk factors for progressive spinal sagittal imbalance. In conclusion, risk factors for progressive spinal sagittal imbalance after THA were weak preoperative ATMS and small SS.

## 1. Introduction

Hip osteoarthritis (OA) is one of the most common musculoskeletal diseases in an aging society [1]. In recent years, it has been recognized that pelvic and spinal alignments affect each other, and many studies have been conducted on spinopelvic alignment in hip OA [2,3,4,5,6]. Patients with hip OA often have hip flexion contracture, pelvic anteversion, and a high sagittal vertical axis (SVA), suggesting spinal sagittal imbalance [7]. As OA progresses and the range of motion of the hip decreases, spinal sagittal imbalance progresses [4,8].

Total hip arthroplasty (THA) is the standard procedure for hip OA, and the number of THAs being performed is increasing [9]. In general, THA reduces flexion contracture of the hip joint, resulting in a retroverted pelvis, decreased SVA, and improved spinal sagittal balance [10,11,12]. However, in some cases, spinal sagittal imbalance with lumbar kyphosis develops in the course after THA. Corrective surgery for lumbar kyphosis, which increases the risk of hip dislocation after THA [13,14,15], can be necessary for these patients. Therefore, it is important to understand the characteristics of the patients with progressive spinal sagittal imbalance after THA; this has not been studied to date. This study aimed to investigate the risk factors for progressive spinal sagittal imbalance in the short-term course after THA.

## 2. Materials and Methods

### 2.1. Participants

**Figure 1:**
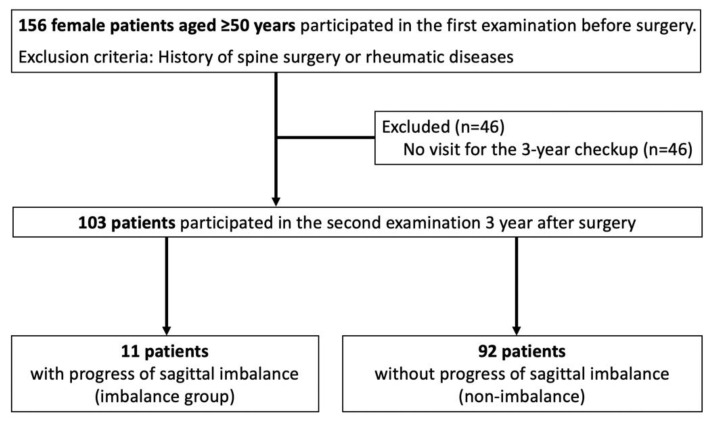
Flowchart describing the selection process of study participants and their assignment to the two groups.

A total of 156 female patients aged ≥50 years who underwent THA for hip OA at our hospital between 2016 and 2019 and agreed to participate in a preoperative examination were included in this study. Patients who had previously undergone spinal surgery or were diagnosed with rheumatic diseases were excluded from the study; 46 patients who did not have the 3 year standing whole-spine radiographs were excluded from the study. Finally, 103 patients were evaluated in this study (Figure 1). Surgical procedures included anterior (*n* = 76), posterior (*n* = 27), unilateral only (*n* = 83), and bilateral simultaneous (*n* = 20) approaches.

### 2.2. Evaluation of the Demographic and Radiographic Parameters of Participants

Clinical information was collected through a retrospective review of a prospectively maintained database. Before the surgery, participants underwent physical measurements, standing whole-spine radiography, and other assessments as described below. We obtained anthropometric measurements, including body height, body weight, and body mass index. Grip power was measured using a TTM dynamometer (Tsutsumi, Tokyo, Japan). The knee extensor muscle strength (KEMS) was measured using a handheld dynamometer (μTas F-1; ANIMA Corp., Tokyo, Japan); KEMS values were divided by the body weight (N/kg). To measure KEMS, subjects were seated in an elevated chair with 90° of knee flexion and their feet above the floor. With the dynamometer placed on the anterior surface of their leg, 10 cm proximal to the malleoli, subjects were instructed to push against the dynamometer by attempting to straighten their knees [16]. The right and left grip power and KEMS were measured once, and the higher strength values were recorded. Abdominal trunk muscle strength (ATMS) was measured using an exercise device for the abdominal trunk muscles (RECORE: Nippon Sigmax Co., Ltd., Tokyo, Japan). As previously described [17], this device enables patients to perform strength measurement or strengthening exercises involving their abdominal trunk muscles in a sitting position without movement of the trunk or load on the spine. A previous study showed that this device had excellent intra- and inter-rater reliabilities to measure ATMS, and strengthening exercises using the device activate and increase diaphragmatic, abdominal, and pelvic floor muscle strength [18]. ATMS was measured twice, and the higher strength values were recorded. Locomotive syndrome, defined as a condition of reduced mobility due to an impairment of a locomotive organ, was assessed using the 25-question Geriatric Locomotive Function Scale (GLFS-25) and a two-step test in each patient [19]. In addition, the functional reach test (FRT) was performed to measure the stability of the patients while reaching forward in the standing position [20]. Other assessments included 10 m walking speed, one-leg standing time, lumbar bone mineral density measured using dual-energy X-ray absorptiometry, and the presence of osteoporotic vertebral fracture evaluated using standing whole-spine radiography. Sagittal spinal alignment was evaluated using whole-spine radiography. Sagittal balance was assessed using the SVA, defined as the distance between the postero-superior corner of S1 and the plumb line drawn from the vertebral midbody of C7 [21]. The following spinopelvic sagittal parameters were measured: lumbar lordosis (LL), pelvic incidence (PI), sacral slope (SS), pelvic tilt (PT), and PI minus LL.

Three years after surgery, participants underwent a follow-up standing whole-spine radiography. Patients were assigned into two groups on the basis of their preoperative and 3 year postoperative SVA values. Patients with a 3 year postoperative SVA value ≥40 mm with an increase ≥30 mm were categorized into the imbalance group, while the others were assigned to the non-imbalance group.

### 2.3. Statistical Analysis

Continuous variables are expressed as the mean ± standard deviation. Between-group differences in continuous variables were examined using the Mann–Whitney U test. Categorical data are expressed as percentages, and comparisons between groups were performed using the chi-squared test. A multiple logistic regression model was used to obtain odds ratios (ORs) with 95% confidence intervals (CIs) to identify the factors associated with progressive spinal sagittal imbalance after THA. Lastly, receiver operating characteristic (ROC) curve analysis was used to determine the optimal cutoff for the occurrence of spinal sagittal imbalance. IBM SPSS Statistics for Macintosh, Version 27.0 (IBM Corp., Armonk, NY, USA) was used for all statistical analyses, with a level of statistical significance of *p* < 0.05.

## 3. Results

A total of 103 patients were included and evaluated in this study. The mean change in SVA was −2.6 mm (range: −103.7 to 156.5 mm). Of the 103 patients, 11 (10.7%) were assigned to the imbalance group, while the other 92 (89.3%) patients were assigned to the non-imbalance group (Figure 1). Preoperatively, the imbalance group had lower ATMS and KEMS values, a shorter FRT distance, and smaller SS and LL values than the non-imbalance group (Table 1). There were no significant differences in the preoperative SVA between the two groups. In the multiple logistic regression analysis using the five items that showed significant differences in the univariate analysis as explanatory variables, preoperative weak ATMS and small SS were significant risk factors for progressive spinal sagittal imbalance after THA (Table 2).

**Figure 2:**
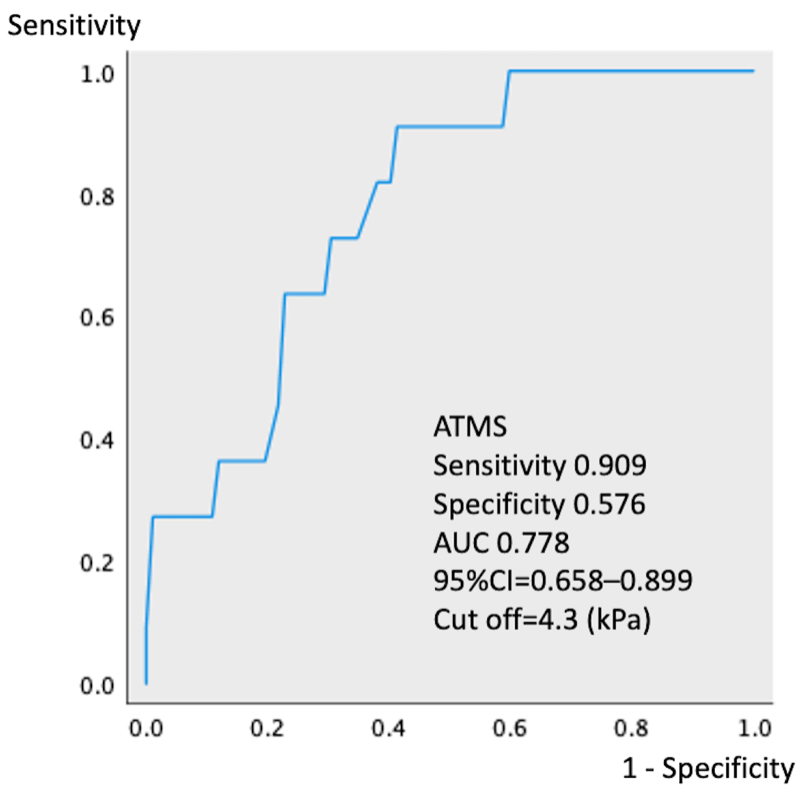
Receiver operating characteristic analysis showing that the cutoff value for abdominal trunk muscle strength is 4.3 kPa (95% confidence interval 0.643–0.909, *p* = 0.004, AUC 0.776). The sensitivity and specificity are 0.909 and 0.576, respectively. ATMS, abdominal trunk muscle strength; AUC, area under the curve; CI, confidence interval.

**Figure 3:**
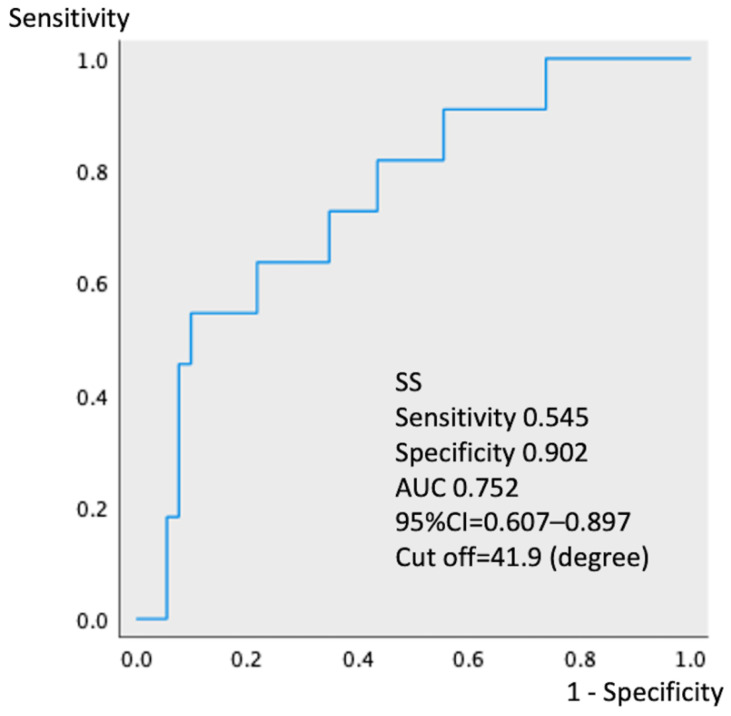
Receiver operating characteristic analysis showing that the cutoff value for sacral slope is 31.9° (95% confidence interval 0.607–0.897, *p* = 0.001, AUC 0.752). The sensitivity and specificity are 0.545 and 0.902, respectively. SS, sacral slope; AUC, area under the curve; CI, confidence interval.

ROC analyses showed that ATMS ≤ 4.3 kPa (Figure 2, 95% CI 0.643–0.909, *p* = 0.004, area under the curve 0.776) and SS ≤ 31.9° (Figure 3, 95% CI 0.607–0.897, *p* = 0.001, area under the curve 0.752) best predicted the progression of sagittal imbalance in the study cohort. The progression rate of sagittal imbalance was significantly higher (*p* = 0.002) in participants with ATMS ≤ 4.3 kPa (20.4%, 10/49) than in those with ATMS > 4.3 kPa (1.9%, 1/54). Similarly, the progression rate of sagittal imbalance was significantly higher (*p* = 0.001) in participants with SS ≤ 31.9° (40.0%, 6/15) than in those with SS > 31.9° (5.7%, 5/88).

## 4. Discussion

This retrospective cohort study with a prospectively maintained database aimed to investigate risk factors associated with progressive spinal sagittal imbalance after THA in middle-aged women. In this study, spinal sagittal imbalance progressed in 11 patients (10.7%) 3 years postoperatively, and multivariate analysis showed that the risk factors were preoperative weak ATMS and small SS. Furthermore, ROC analysis showed that ATMS ≤ 4.3 kPa and SS ≤ 31.9° were associated with spinal sagittal imbalance and may be useful predictors of progressive spinal sagittal imbalance before THA. Although many reports evaluated spinopelvic alignment immediately after THA [10,11,12,22,23,24,25,26], to our knowledge, no studies have reported the risk of spinal sagittal imbalance in the long-term course after THA. Spinal sagittal imbalance after THA may lead to a demand for corrective surgery. Correction of lumbar kyphosis changes the pelvic tilt, resulting in an increased risk of dislocation of the artificial hip [13,14,15]. Therefore, assessing the trunk function and spinopelvic alignment before THA is important to predict the progression of spinal sagittal imbalance according to the results of this study.

The results of this study suggested that a weak ATMS is a risk factor for spinal sagittal imbalance after THA. The relationship between trunk muscle strength and spinal sagittal balance has been reported in the past, and these studies concluded that weak back muscles are associated with sagittal imbalance [27,28,29,30]. A previous study showed that the device used in this study (RECORE) could quantify ATMS, and strengthening exercises using the device increased ATMS and activated the abdominal, diaphragmatic, and pelvic floor muscles [18]. Muscle contraction during the ATMS measurement or strengthening exercise was comparable to that involved during abdominal bracing with the abdominal and paraspinal muscles activated [31]. The core can be described as a muscular box whose anterior wall is represented by the abdominal muscles, with the posterior wall represented by the paraspinal muscles, the roof represented by the diaphragm, and the floor represented by the pelvic floor muscles [32]. The contraction of the diaphragm increases intra-abdominal pressure, thus contributing to spinal stability [32]. ATMS is created through the coordinated contraction of trunk muscles comprising the muscular box. This muscle contraction creates a semirigid cylinder located anterior to the spinal column that can support the spinal column from the front and prevent a progression of sagittal spinal imbalance in the lower thoracic or lumbar spines. Although several studies have described the contribution of these anterior trunk muscles to spinal stability [32,33], there have been no previous reports of ATMS being associated with spinal sagittal imbalance. On the basis of the study’s results, the functional assessment of the trunk, including ATMS measurements in patients with hip OA, is important for predicting progressive spinal sagittal imbalance after THA, which is clinically problematic. The device allows for easy and safe ATMS measurements [34], and the ATMS measurement has great potential to be used in assessing trunk dysfunctions related to the future progression of spinal sagittal imbalance after THA.

**Figure 4:**
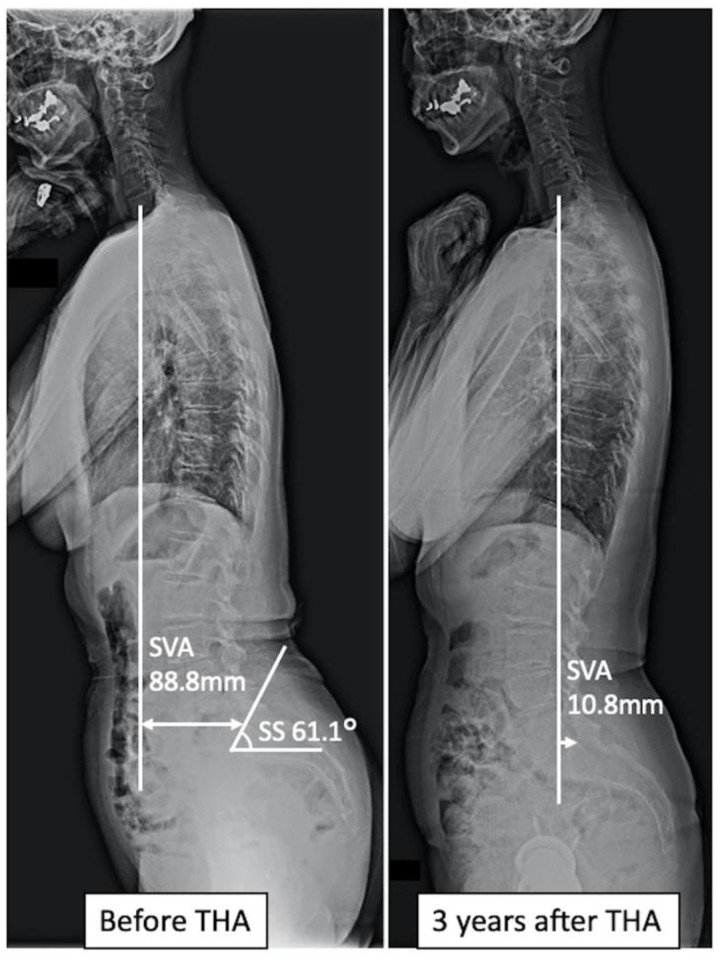
A case with improved spinal sagittal balance after total hip arthroplasty. Preoperatively, the sacral slope, sagittal vertical axis (SVA), and abdominal trunk muscle strength were 61.1°, 88.8 mm, and 5.2 kPa, respectively. Postoperatively, SVA decreased to 10.8 mm.

**Figure 5:**
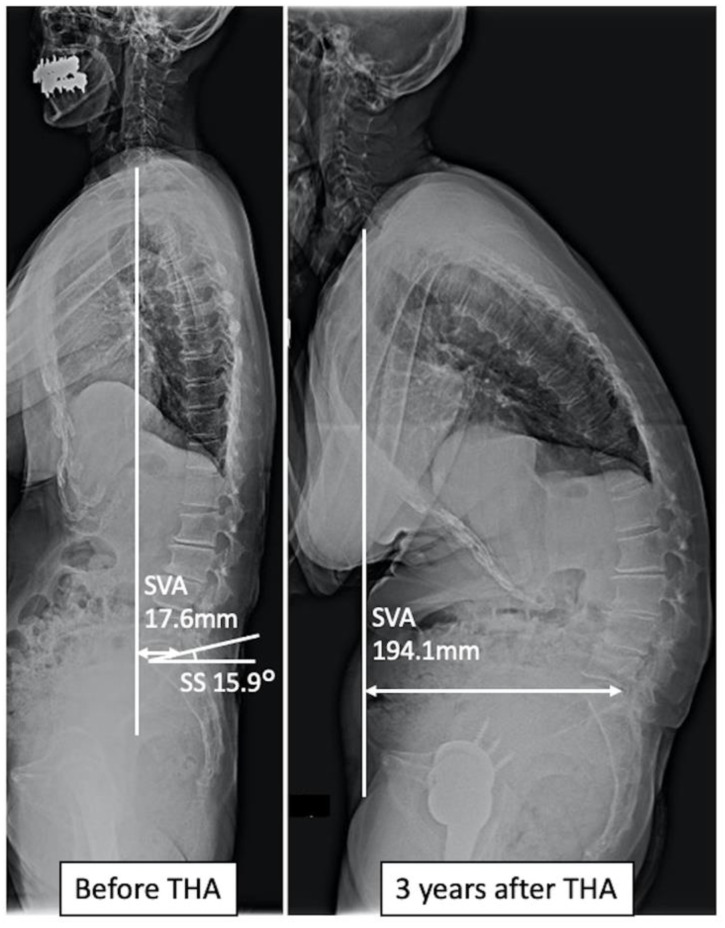
A case of progressive spinal sagittal imbalance after total hip arthroplasty. Preoperatively, the sacral slope, sagittal vertical axis (SVA), and abdominal trunk muscle strength were 15.9°, 17.6 mm, and 3.2 kPa, respectively. Postoperatively, SVA increased to 194.1 mm.

This study showed that a small SS causes spinal sagittal imbalance 3 years after THA in female patients. Patients with hip OA usually have increased SVA due to anterior pelvic tilt caused by hip flexion contracture [11]. The mean values of SS and PT of Japanese women in their 60s were 31° and 18°, respectively [35]. In contrast, those in the non-imbalance group in this study had values of 42° and 14°, respectively, indicating that the pelvis was tilted anteriorly. On the other hand, the mean values of SS and PT of the imbalance group were 32° and 18°, respectively, similar to those of the general population of the same age, despite the presence of hip OA. In cases of anterior pelvic tilt due to flexion contracture, as in the non-imbalance group in this study, SVA decreased immediately after THA because flexion contracture improved, and the pelvis rotated backward [10,11,12] (Figure 4). However, since patients with a small SS have a smaller capacity for retroversion of the pelvis [33], their spinal sagittal balance did not improve. In addition, patients with a small SS are less capable of compensating for the progressive decrease in lumbar lordosis over time, making it difficult for them to maintain spinal sagittal balance (Figure 5). These results may explain why spinal sagittal imbalance is more likely to progress in patients with a small SS in the long term after THA.

This study had several limitations. First, the back muscle strength, considered a risk factor for spinal sagittal imbalance, was not measured. Thus, it is impossible to compare the effects of ATMS and back muscle strength on the progression of sagittal imbalance. Second, the spinopelvic alignment was not measured immediately after surgery. Therefore, it is unclear to what extent the immediate postoperative changes and the 3 year course after surgery affect sagittal balance. Third, since this study did not include nonsurgical patients, it is not certain whether the change in sagittal balance was the effect of surgery or a natural course.

## 5. Conclusions

Spinal sagittal imbalance progressed in approximately 10% of patients 3 years after THA. Preoperative weak ATMS and small SS were risk factors for progressive spinal sagittal imbalance in the short-term course after THA in female patients. Preoperative assessments of trunk function and spinopelvic alignment are important predictors of progressive sagittal imbalance after THA.

## Figures and Tables

**Table 1:** Demographic and radiographic parameters of 103 patients.

	Imbalance Group	Non-Imbalance Group	*p*-Value
No. of subjects	11	92	
Age (years), mean ± SD	64.7 ± 9.3	63.8 ± 8.7	0.902
BMI (kg/m^2^), mean ± SD	25.0 ± 7.7	23.1 ± 3.8	0.860
Bilateral simultaneous THA, No. (%)	2 (18.2)	18 (19.6)	0.946
Anterior approach, No. (%)	9 (81.8)	67 (72.8)	0.48
Grip strength (kg), mean ± SD	20.5 ± 4.9	21.1 ± 4.8	0.435
KEMS (N/kg), mean ± SD	2.9 ± 1.4	3.9 ± 1.2	0.026
ATMS (kPa), mean ± SD	2.5 ± 1.6	5.2 ± 2.9	0.013
FRT (cm), mean ± SD	26.1 ± 6.4	30.9 ± 7.1	0.019
Walking speed (m/s), mean ± SD	0.8 ± 0.4	0.9 ± 0.2	0.359
1-leg standing time (s), mean ± SD	25.7 ± 21.4	36.7 ± 22.9	0.142
2-step score (point), mean ± SD	0.8 ± 0.3	1.0 ± 0.3	0.342
GLFS-25 score (point), mean ± SD	52.6 ± 18.7	43.8 ± 18.5	0.173
L-BMD (g/cm^2^), mean ± SD	1.2 ± 0.2	1.1 ± 0.2	0.275
Presence of OVF before THA, No. (%)	1 (9.0)	11 (12.0)	0.624
Occurrence of OVF after THA, No. (%)	0 (0.0)	2 (2.1)	0.797
SVA (mm), mean ± SD	47.9 ± 26.1	45.3 ± 47.2	0.665
LL (degrees), mean ± SD	40.1 ± 18.7	53.3 ± 17.9	0.019
PI (degrees), mean ± SD	51.0 ± 12.6	57.2 ± 11.6	0.075
SS (degrees), mean ± SD	32.7 ± 10.2	42.7 ± 12.4	0.006
PT (degrees), mean ± SD	18.3 ± 8.9	14.4 ± 11.4	0.175
PI-LL (degrees), mean ± SD	10.9 ± 15.2	3.9 ± 16.9	0.172

ATMS, abdominal trunk muscle strength; BMI, body mass index; FRT, functional reach test; GLFS-25, 25-question Geriatric Locomotive Function Scale; KEMS, knee extensor muscle strength; L-BMD, bone mineral density of the lumbar spine; LL, lumbar lordosis; OVF, osteoporotic vertebral fracture; PI, pelvic incidence; PT, pelvic tilt; SD, standard deviation; SVA, sagittal vertical axis; SS, sacral slope; THA, total hip arthroplasty.

**Table 2:** Multiple logistic regression analysis of factors associated with progressive spinal sagittal imbalance.

	OR	*p*-Value	95% CI
ATMS (kPa)	0.504	0.007	0.307–0.827
SS (degrees)	0.924	0.005	0.875–0.976

ATMS, abdominal trunk muscle strength; CI, confidence interval; OR; odds ratio; SS, sacral slope.

## Data Availability

The datasets used and analyzed during the current study are available from the corresponding author on reasonable request. The data are not publicly available due to privacy or ethical restrictions.
